# Numb cheek syndrome in breast cancer: a case report

**DOI:** 10.3389/fonc.2024.1349073

**Published:** 2024-03-11

**Authors:** Zhibin Tan, Si Ying Tan

**Affiliations:** ^1^ Department of Neurology, National Neuroscience Institute, Singapore, Singapore; ^2^ Neuroscience Academic Clinical Programme, Duke-NUS Medical School, Singapore, Singapore; ^3^ SingHealth Duke-NUS Breast Centre, SingHealth, Singapore, Singapore; ^4^ Department of Breast Surgery, Division of Surgery and Surgical Oncology, Singapore General Hospital, Singapore, Singapore; ^5^ Department of Breast Surgery, Division of Surgery and Surgical Oncology, National Cancer Centre Singapore, Singapore, Singapore

**Keywords:** breast cancer, malignancy, numb cheek, maxillary, trigeminal, neuropathy

## Abstract

**Background:**

Numb cheek syndrome, a rare corollary of numb chin syndrome, is due to infra-orbital neuropathy. It can occur in association with an underlying malignancy, which can cause neuropathy by direct malignant nerve infiltration or via a paraneoplastic mechanism. Although numb cheek syndrome has been reported in association with a variety of cancers, it has previously not been reported in association with breast cancer. We report a case of left breast cancer presenting with left numb cheek syndrome.

**Case presentation:**

A 65-year-old woman presented to the Neurology clinic with a 7-month history of left cheek numbness and occasional cheek tenderness. Examination revealed slightly diminished pin-prick sensation in the left cheek and a vaguely palpable left breast lump. A magnetic resonance imaging scan of the brain showed abnormal enhancement of the left maxillary nerve at the foramen rotundum, but cerebrospinal fluid analysis was normal. Mammography, ultrasound scans, and core biopsy of the left breast confirmed the diagnosis of invasive left breast carcinoma (estrogen and progesterone receptor negative, c-erb-B2 equivocal, fluorescence *in-situ* hybridization negative). There was no evidence of distant metastases on computed tomography and bone scintigraphy scans. The patient underwent neoadjuvant chemotherapy (4 cycles of doxorubicin and cyclophosphamide, followed by 4 cycles of paclitaxel and carboplatin), and left breast wide excision and sentinel lymph node biopsy, and a repeat magnetic resonance imaging scan performed 2 months after surgical resection showed resolution of the left maxillary nerve enhancement. The patient’s left numb cheek symptoms improved over a course of 5 months after cancer resection but did not completely resolve.

**Conclusions:**

Our case represents the first reported left numb cheek syndrome in association with breast cancer, due to maxillary neuropathy without any discrete mass or compressive cause. To avoid delays in diagnosing malignancy, physicians and surgeons should be aware that numb cheek syndrome can occur in association with an underlying malignancy, and that breast cancer should be counted amongst the possibilities.

## 1 Introduction

Numb chin syndrome, a manifestation of mental neuropathy, can be associated with a variety of benign causes, but it can also uncommonly be a presentation of malignant disease (1). Numb cheek syndrome is an even rarer corollary phenomenon, caused by infra-orbital or maxillary neuropathy, which can also be associated with malignancy (2). While numb chin syndrome has been well described in association with breast cancer (3), numb cheek syndrome has not.

Herein, we present a case of left breast cancer presenting with left numb cheek syndrome.

## 2 Case presentation

A 65-year-old woman with a past medical history of hyperlipidemia and benign hyperplastic colonic polyps presented to the Neurology specialist outpatient clinic for a 7-month history of left cheek numbness, having been referred by her General Practitioner. Her numbness was a negative sensory symptom, which she described as a sensation of cheek swelling even though it appeared normal in the mirror, accompanied by diminished sensation to touch. There was also occasional tenderness on palpation of her left cheek. There were otherwise no symptoms over her left forehead, left mandible, or any part of the right face. The patient denied having any constitutional symptoms, but she had a family history of breast cancer, with her sister having been diagnosed with left breast invasive ductal carcinoma (estrogen and progesterone receptor positive, c-erb-B2 negative) at the age of 44-years-old. The patient was not taking any medications prior to presentation.

On examination, pin-prick sensation was 20% diminished over the left cheek compared to the right cheek. The area of numbness spanned the skin over the left maxilla, left lower eyelid, and left philtrum. The rest of the neurological examination, including examination of the other cranial nerves and of the long tracts, was normal. No asymmetry of motor power in trigeminal nerve-innervated muscles (pterygoids and masseter) was detected. Attention was paid to the skin and buccal mucosa overlying the area of numbness, but no lesions were seen.

**Figure 1:**
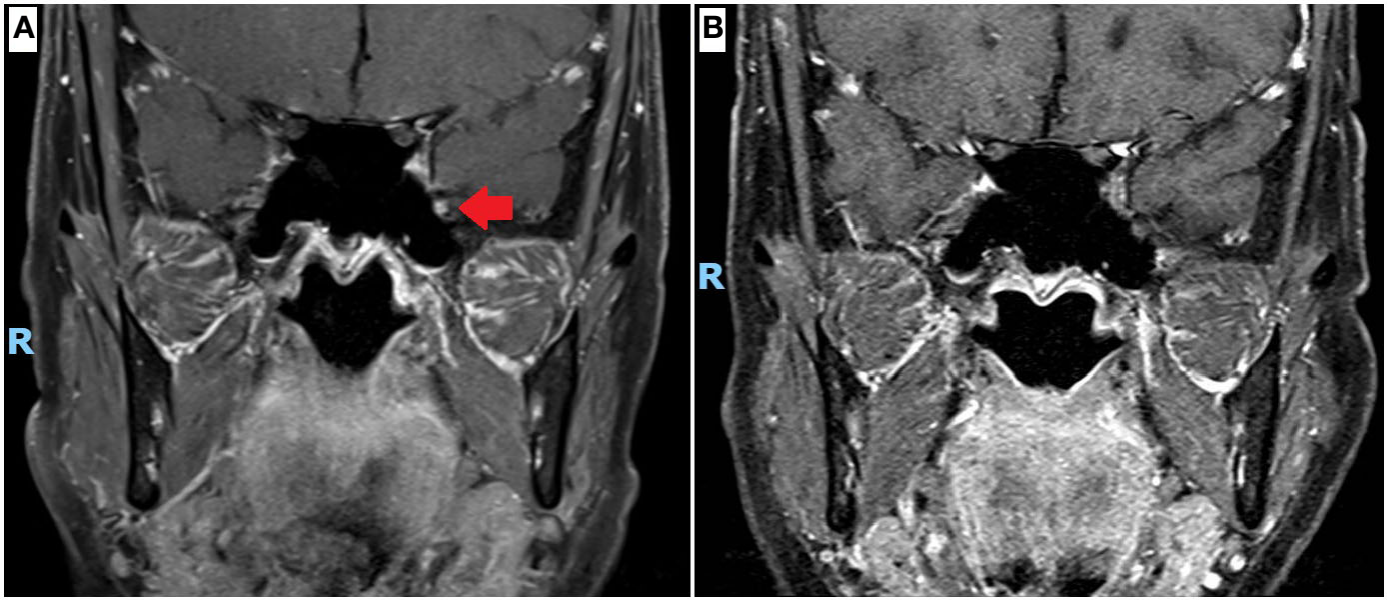
Magnetic resonance imaging (MRI) scan of the cranial nerves. **(A)** Coronal projection, T1 with gadolinium contrast at 12 months since onset of left numb cheek, showing left maxillary nerve enhancement at the foramen rotundum (red arrow). **(B)** Coronal projection, T1 with gadolinium contrast at 26 months since onset, performed after completion of neoadjuvant chemotherapy and surgical resection of breast cancer, showing resolution of left maxillary nerve enhancement.

As the symptoms were mild and non-disabling, no medications were prescribed at first presentation. A magnetic resonance imaging (MRI) scan of the brain and cranial nerves was performed approximately 1 year from onset of symptoms (5 months from presentation), revealing a non-compressive, T2-hyperintense, T1 isotense, gadolinium-enhancing lesion of the left maxillary nerve in the foramen rotundum (**Figure 1**). The rest of the brain parenchyma was unremarkable.

Blood tests, including a panel of onconeural antibodies, were unremarkable (**Table 1**). A lumbar puncture was performed 13 months from onset, showing a normal opening pressure of 16.5cmH_2_O. Cerebrospinal fluid (CSF) analysis showed normal CSF cell counts (CSF red blood cell count 0/μL, CSF white blood cell count 1/μL), normal CSF protein level (0.29g/L), and a normal CSF-to-serum glucose ratio of 0.6 (CSF glucose 3.5mmol/L, paired serum glucose 5.6mmol/L). A panel of CSF microbiological and cytometric tests were unyielding (**Table 1**). CSF cytology did not find any malignant cells. CSF onconeural antibodies were not tested, due to institutional limitations on test availability.

**Table 1:** Summary of blood and cerebrospinal fluid investigations.

Tests on serum/blood	
Infectious/Para-infectious	*Hepatitis B* surface antigen *Hepatitis C* antibody
Inflammatory/autoimmune	CRP, ESR Anti-nuclear antibody Anti-double stranded DNA antibody ANCA, anti-MPO antibody, anti-PR3 antibody Extractable nuclear antigen profile (anti-Smith, anti-ribonucleoprotein, anti-Ro, anti-La, anti-Scl 70, anti-Jo 1 antibodies)
Malignancy	Onconeural antibodies (Hu, Yo, Ri, CRMP5, amphiphysin, PNMA2/Ta, recoverin, SOX1, titin, zic4, GAD65, Tr)
Tests on CSF	
Infectious/Para-infectious	Gram stain, culture Acid fast bacilli smear and culture *Mycobacterium tuberculosis* complex DNA amplification Fungal microscopy and culture VDRL PCR tests (*Escherichia coli, Hemophilus influenzae, Listeria monocytogenes, Neisseria meningitidis, Streptococcus agalactiae, Streptococcus pneumoniae*, CMV*, Enterovirus*, HSV1, HSV2, HHV6*, Human parechovirus*, VZV*, Toxoplasma, Cryptococcus*)
Malignancy	Flow cytometry Cytology

All negative or normal. ANCA, anti-neutrophil cytoplasmic antibody; CRMP5, collapsin response mediator protein 5; CRP, c-reactive protein; CSF, cerebrospinal fluid; DNA, deoxyribonucleic acid; ESR, erythrocyte sedimentation rate; GAD65, glutamic acid decarboxylase 65-kilodalton isoform; HHV6, human herpes virus 6; HSV, herpes simplex virus; LGI1, leucine-rich glioma inactivated 1; MPO, myeloperoxidase; PCR, polymerase chain reaction; PNMA2, paraneoplastic antigen Ma2; PR3, proteinase 3; SOX1, Sry-like high mobility group box 1; VDRL, venereal disease research laboratory; VZV, varicella zoster virus.

**Figure 2:**
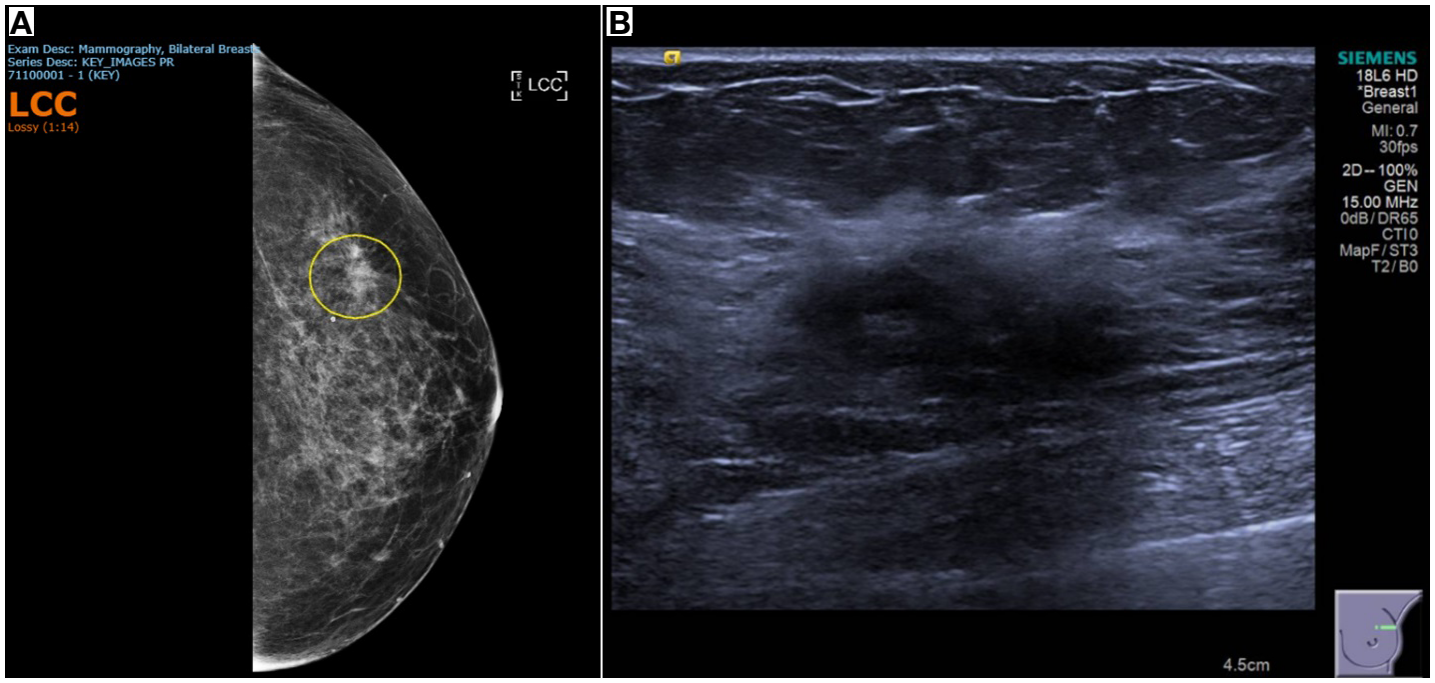
Breast imaging showing tissue that was subsequently proven to be triple-negative ductal carcinoma with apocrine change. **(A)** Mammography of the left breast showing heterogeneously dense breast stroma, with an area of distortion in the left breast upper outer quadrant, and a density, persisting on cone compression. **(B)** 28 x 12 x 21mm left breast 1 o’clock vague area of ill-defined hypoechoic tissue, associated with mild distortion and posterior shadowing.

A computed tomography (CT) scan of the chest, abdomen, and pelvis did not find any evidence of malignancy or metastasis. Mammography and ultrasound scans of the breasts, performed 14 months after the onset of left numb cheek, showed a 6 × 10 × 6mm area of left breast upper outer quadrant hypoechoic tissue, suspicious for malignancy (**Figure 2**). With knowledge of the scan findings, a vague left breast nodule was palpable on examination. Core biopsy provided histological confirmation of left breast triple-negative ductal carcinoma with apocrine change (estrogen and progesterone receptor negative, c-erb-B2 equivocal, fluorescence *in-situ* hybridization negative). A technetium bone scintigraphy scan did not show any evidence of bony metastases.

Following recommendations made at a multi-disciplinary tumor board meeting, the patient underwent neoadjuvant chemotherapy (4 cycles of doxorubicin and cyclophosphamide, followed by 4 cycles of paclitaxel and carboplatin) over the course of 7 months (17 to 23 months since left numb cheek onset). She then underwent left breast wide excision and sentinel lymph node biopsy 24 months after onset, with histology showing residual ductal carcinoma *in-situ* 1mm away from the closest resection margin. Two sentinel lymph nodes were negative for malignancy.

Prior to the commencement of chemotherapy, the patient underwent a repeat MRI scan of the brain and cranial nerves (16 months after onset). There were no changes from the original MRI scan (12 months after onset); with persistent enhancement of the left trigeminal nerve at the foramen rotundum noted. After completion of neoadjuvant chemotherapy and surgical resection, a repeat MRI scan (26 months after onset) showed resolution of the left trigeminal nerve enhancement (**Figure 1**).

Clinically, the patient’s symptom of left numb cheek with occasional tenderness remained persistent throughout the course of neoadjuvant chemotherapy. Only after surgical resection did she report a gradual improvement in cheek numbness and pain. At follow-up 5 months after surgical resection, the patient reported only minimal numbness over the left philtrum. Mammography at 4 months (scheduled as part of national cancer screening that the patient was subsequently enrolled in) after surgical resection did not show any evidence of malignancy.

## 3 Discussion

Breast cancer is the most commonly diagnosed cancer worldwide, with approximately 2.3 million new cases and 685,000 deaths per year (4). Early diagnosis is imperative in the management of breast cancer, given that those with more advanced disease at diagnosis have worse prognoses, with 5-year survival rate declining from 97% for those with stage 1 disease to 48% for those with stage 4 disease (5). Despite the high prevalence and urgency of diagnosis, breast cancer can be easily missed in a patient with the unusual and seemingly benign presentation of numb cheek syndrome. This is illustrated by our case, in which the first MRI scan of the brain was arranged with low priority, and performed 5 months after initial presentation, leading to a significant delay in detection of the maxillary nerve abnormality which turned out to be the sole extra-mammary site of disease. We therefore wish to raise awareness amongst physicians and surgeons that a presentation with unilateral numb cheek can be associated with not only malignancy in general, but also specifically breast cancer.

Prior reports of malignant numb cheek syndrome have been in association with extra-mammary cancers, such as recurrent basal cell epithelioma, newly diagnosed squamous cell carcinoma of the face, metastatic prostate adenocarcinoma, and anaplastic small cell carcinoma of the lung with anti-Hu antibody (2, 6, 7). Trigeminal neuropathy, when reported in association with breast cancer, has been secondary to mechanical compression or radiologically visible tumor infiltration by an adjacent metastatic mass. The sites of involvement reported have been more proximal segments of the trigeminal nerve, from its pontine origin to the Gasserian ganglion, resulting in wider areas of hemifacial dysesthesia or pain (i.e., trigeminal neuralgia) (8–10). To our knowledge, our case represents the first case of a true numb cheek syndrome associated with breast cancer, with symptoms and signs confined to the territory of the second division of the trigeminal nerve (i.e., maxillary nerve), with no discrete mass or compressive cause found. Numb cheek syndrome is traditionally localized to an infra-orbital neuropathy, but our patient with maxillary neuropathy at the foramen rotundum presented with numb cheek syndrome because the cutaneous distribution of the infra-orbital nerve is coterminous with the field of the maxillary nerve (2).

In our patient, the unyielding CSF tests and lack of explanation for the abnormal maxillary nerve signal on MRI led to our search for distant tumors. We favored a CT scan of her thorax, abdomen, and pelvis because it was more readily available, but a Positron Emission Tomography (PET) scan was also being considered. Ultimately, a PET scan was not performed for our patient because application of our national age-appropriate cancer-screening guidelines (recommending mammography and ultrasound breast scans) had already identified a plausible cause of her maxillary nerve abnormality.

Interestingly, our patient’s CT scan report did not detect the presence of any underlying breast tumor, even on retrospective review. While existing guidelines do not recommend the use of CT scans for breast cancer screening due to scant evidence of benefit and higher radiation exposure, there is some evidence that CT scans with cuts as fine as 1.25mm may be comparable to mammography in sensitivity (11, 12). For our patient, breast cancer may have gone undetected on CT scan because the slice thickness was 3mm, possibly causing a 6 × 10 × 6mm lesion to appear small and non-specific.

This was also an opportune revisitation of the pathophysiology of numb cheek syndrome. Left maxillary neuropathy in our patient may have been the result of hematogenous spread, leading to leptomeningeal metastasis and/or direct nerve infiltration. This possibility is supported by previous reports of breast cancer with metastatic involvement of other nerves (13). Furthermore, triple-negative breast cancer, like that of our patient, has also been reported to have a preponderance for early nervous system metastases, with approximately one-third of cases eventually developing brain metastases (14, 15). The lack of malignant cells in our patient’s CSF cytology does not rule out leptomeningeal metastasis or direct parenchymal infiltration, because CSF cytology has suboptimal sensitivity for both kinds of metastases (16, 17). However, when combined with the normal CSF protein concentration and lack of CSF pleocytosis in our patient, leptomeningeal metastasis becomes less likely, given that the majority of patients with leptomeningeal metastasis will have abnormalities in both CSF cell counts and protein concentrations (18).

A paraneoplastic mechanism also remains a viable explanation. Paraneoplastic neuropathy is thought to be the result of onconeural antibodies targeting cross-reacting intracellular antigens of neuronal and tumoral tissues, with T-cell cytotoxicity likely playing a role in neuronal injury (19). Although our patient’s onconeural antibody tests returned negative, existing panels are unlikely to be exhaustive. Given that paraneoplastic neuropathy can lead to long-term deficits with or without anti-tumor treatment and even immunotherapy, our patient’s improved but persistent symptoms after neoadjuvant chemotherapy and surgical tumor resection do not rule out a paraneoplastic mechanism (20).

Other pathologic mechanisms, such as mechanical nerve compression and adjacent osseous involvement, have been suggested for numb cheek or chin syndrome (1), but these were not observed in our patient, given the absence of adjacent masses or bony metastases on her scans.

## 4 Conclusions

We presented a case of numb cheek syndrome in association with breast cancer, hoping to raise awareness that numb cheek syndrome can occur in association with malignancy, where we postulate that it can be due metastasis or paraneoplastic neuropathy involving the infra-orbital or maxillary nerve, even without a discrete adjacent mass causing nerve compression. Although not previously reported until now, breast cancer should be included in the list of malignancies that can be associated with numb cheek syndrome.

## Data availability statement

The original contributions presented in the study are included in the article. Further inquiries can be directed to the corresponding author.

## Ethics statement

Ethical approval was not required for this case report involving one human subject in accordance with the local legislation and institutional requirements. Written informed consent was obtained from the individual(s) for the publication of any potentially identifiable images or data included in this article.

## Author contributions

ZT: Conceptualization, Data curation, Formal analysis, Investigation, Methodology, Validation, Writing – original draft, Writing – review & editing. ST: Conceptualization, Data curation, Formal analysis, Investigation, Methodology, Writing – review & editing.
